# The Expression of PD-L1 and B7-H4 in Thymic Epithelial Tumor and Its Relationship With Tumor Immune-Infiltrating Cells

**DOI:** 10.3389/fonc.2021.662010

**Published:** 2021-07-08

**Authors:** Xiaotian Yan, Jie Feng, Bo Hong, Yun Qian

**Affiliations:** ^1^ Department of Clinical Laboratory, The Second Affiliated Hospital, Zhejiang University School of Medicine, Hangzhou, China; ^2^ Department of Blood Transfusion, The Second Affiliated Hospital, Zhejiang University School of Medicine, Hangzhou, China; ^3^ Department of Pathology, The Second Affiliated Hospital, Zhejiang University School of Medicine, Hangzhou, China

**Keywords:** thymic epithelial tumor, immune checkpoint inhibitors, PD-L1, B7-H4, tumor immune-infiltrating cells, tumor microenvironment, immunohistochemistry, prognosis

## Abstract

**Background:**

PD-L1 and B7-H4 have been reported to be expressed in various malignancies and are considered as promising prognostic factors and potential immunotherapy targets.

**Methods:**

We analyzed the correlation between the expression of PD-L1 and B7-H4 transcriptomes and clinicopathological characteristics in 121 TET patients from The Cancer Genome Atlas (TCGA) database. The immune-infiltration levels in the TET microenvironment were estimated using ssGSEA and quanTiseq algorithms. We collected 80 TET cases from 2008 to 2015. PD-L1、B7-H4、FOXP3 and CD163 protein expression in tumor tissues were detected by immunohistochemistry.

**Results:**

TCGA database showed PD-L1 mRNA levels can predict the OS (P = 0.018) and DFS (P = 0.033) of TET patients. B7-H4 mRNA levels were positively related to the World Health Organization (WHO) pathological classification (P = 0.003) but not correlated with patient prognosis. Immune infiltration analysis showed PD-L1 is positively correlated with Tregs and M2 macrophages, B7-H4 is positively correlated with Tregs. Patients with high PD-L1 and Tregs or M2 macrophages, high B7-H4 and Tregs had a worse prognosis. Immunohistochemistry showed PD-L1 expression was positively correlated with the WHO pathological classification and Masaoka stage (P = 0.025, 0.003) and high PD-L1 expression can predict the poor OS of patients (P = 0.043); B7-H4 was also positively correlated with WHO pathological classification and Masaoka stage (P = 0.036, 0.049). However, B7-H4 expression did not correlate with patient prognosis. Evaluation of co-expression patterns showed TET patients with a high-grade WHO pathological classification harbored a 44.4% co-expression of PD-L1 and B7-H4. In addition, we found the expression level of PD-L1 is positively correlated with FOXP3 and CD163 (P = 0.004, P = 0.029) and B7-H4 is positively correlated with FOXP3 (P = 0.037). High PD-L1 combined with High FOXP3 and High CD163, High B7-H4 combined with High FOXP3 can be used to predict the poor prognosis of TET patients (P = 0.026, 0.031, 0.028, respectively).

**Conclusion:**

PD-L1 and B7-H4 were related to the aggressiveness of TET and their expression level can indicate the suppressive immune microenvironment. Combined with FOXP3 and CD163, PD-L1 and B7-H4 can indicate a poor prognosis of TET.

## Introduction

Thymic epithelial tumor (TET) is a rare tumor type that relies on surgical resection and is typically associated with a good prognosis (1). However, some patients with advanced or metastatic disease cannot undergo resection and can only be treated with radiotherapy and chemotherapy. These patients are prone to relapse and have a poor prognosis. The recent development of immunotherapy targeting immun-checkpoint inhibitors has succeeded in many types of solid tumors and also applied in clinical trial of thymic carcinoma (2, 3). However, conflicting results have been reported (4, 5). Therefore, it is necessary to evaluate the expression of immunosuppressive molecules and its clinical significance in TET patients, which may be helpful to guide the selection and improvement of effective immunotherapy.

In recent years, programmed death ligand 1 (PD-L1) has gained much attention, which can inhibit T cell responses by binding to programmed cell death protein 1 (PD-1) on the surface of tumor cells and promote tumor cells to evade immune surveillance (2). Immune checkpoint inhibitors targeting PD-L1 have been shown to have good efficacy in melanoma and non-small cell lung cancer (6–8). Previous studies have shown that most TET patients express PD-L1 (9, 10), and PD-L1 expression is related to the Masaoka stage and World Health Organization (WHO) pathological classification of TET (11–13), but there is still controversy regarding the prognostic significance of PD-L1 (11, 12, 14).

As with PD-L1, the protein B7-H4 also belongs to the B7 family. B7-H4 is also a negative co-stimulatory molecule, which can promote tumor cells to escape immune surveillance and can play an essential role in the formation of the tumor microenvironment. Studies have shown that B7-H4 has low expression in normal tissues but high expression in pancreatic cancer (15), ovarian cancer (16), breast cancer (17), and other malignant tumors and is closely related to the occurrence and development of tumors. However, there are few studies exploring the expression of B7-H4 in TET.

More and more studies have shown that the tumor microenvironment (TME), especially the characteristics of tumor-infiltrating immune cells (TIICs) in TME is related to the occurrence and progression of cancer (18, 19). The type and density of TIICs can not only predict the survival of patients, but also reflect the tumor response to therapy (20). Therefore, TIICs have broad prospects as clinical biomarkers for malignant tumor. Among them, it should be noted that suppressor cells that inhibit tumor activity can affect tumor progression and drug efficacy. FOXP3^+^ T regulatory cells and CD163^+^ M2 macrophages are representative suppressor cells and have been reported as negative prognostic factors in several solid tumors (21, 22). However, few studies have investigated the tumor immune invasion and the prognostic role of TIICs in TET. Due to the complexity of tumor immune response, it is limited to use a single biomarker to predict the patient’s response. Therefore, the combination of immunosuppressive molecules and immune infiltrating cells in TME can be used to assess tumor conditions, prompt the prognosis of patients, and even guide the personalized customization of immunotherapy.

In this study, we evaluated the expression patterns and clinical significance of PD-L1 and B7-H4 in TET patients, as well as their correlation with tumor immune-infiltrating cells and combined prognostic significance. We aimed to provide new insights into the development of clinical immunotherapy and prognostic factors of TET.

## Materials and Methods

### Transcriptome Data Analysis in TCGA and GTEx Database

We used the R language package to download the published thymic epithelial tumor (TET) transcriptome data set from The Cancer Genome Atlas (TCGA) and the corresponding clinical information of the assessed tumor patients. We obtained matched normal thymus tissues collected from the Genotype-Tissue Expression (GTEx) database. A total of 121 TET tissues and 444 normal tissues were analyzed for PD-L1 and B7-H4 mRNA expression. mRNA expression data were further normalized using upper quartile FPKM (FPKM-UQ) and log_2_ transformed before analysis as described in TCGA and GTEx website.

### Immune Infiltration Analysis Based on Single-Sample Geneset Enrichment Analysis and Tumor Immune Estimation Resource

ssGSEA is a deconvolution algorithm that evaluates the level of immune cell infiltration in a sample based on the expression level of immune cell-specific marker genes (23). We used the R package named “GSVA” to perform ssGSEA analysis of the enrichment scores of tumor immune cells in TET patients from the TCGA dataset. The following 24 types of immune cells were obtained: activated dendritic cells (aDC), B cells, CD8+ T cells, Cytotoxic cells, dendritic cells (DC), eosinopoils, immature dendritic cells (iDC), macrophages, mast cell, neutrophils, NK CD56 bright cells, NK CD56dim cells, natural killer cells (NK cells), plasmacytoid dendritic cells (pDC), T cells, T helper cells, central memory T cells (Tcm), effector memory T cells (Tem), follicular helper T cells (TFH), Tgd, type-1 T helper cells (Th1), type-17 T helper cells (Th17), type-2 T helper cells (Th2) and T regulatory cells (Tregs).

TIMER2.0 (http://timer.cistrome.org/) online tool was used to analyze the relationship between tumor gene expression and immune infiltration (24). We analyzed the correlation of PD-L1 and B7-H4 with the infiltration level of tumor immune-infiltrating cells, including Tregs and M2 macrophages, *via* Gene Module of TIMER2.0 using quanTiseq algorithm. We also used Outcome Module of TIMER2.0 to evaluate the prognosis value of the PD-L1 and B7-H4 expression combined with tumor immune-infiltrating cells in TET.

### Patient and Tissue Specimens

The study followed up 80 TET patients who were treated at the Department of Thoracic Surgery in the Second Affiliated Hospital of Zhejiang University School of Medicine from 2008 to 2015. All patients were primary TET and received surgical resection. Patients who were pathologically diagnosed as thymic cancer after surgery received radiotherapy. The average follow-up time was 79 months (range = 12–156 months). This study followed the ethical guidelines of the Declaration of Helsinki and was approved by Ethics Committee of the Second Affiliated Hospital of Zhejiang University School of Medicine. All of the patients signed an informed consent form.

### Immunohistochemistry

Immunohistochemical staining was performed using a two-step EnVision™ method (Dako, Glostrup, Denmark) as previously described (25). The formalin-fixed and paraffin-embedded thymus tumor tissues were collected and cut into 5 μm-serial sections. The sections were dewaxed with xylene and rehydrated through a graded alcohol series. Endogenous peroxidase activity was blocked using 0.3% hydrogen peroxide solution for 35 min at room temperature, and antigen extraction was performed at 100°C for 30 min in citrate buffer (10 mmol/L; pH 6.0). Washing with phosphate-buffered saline (PBS) for 5 min three times, sections were incubated with 10% normal goat serum to block non-specific binding. Sections were then incubated with a rabbit anti-human B7-H4 monoclonal antibody (1:400 dilution; clone number EP1165; Abcam, MA, USA), a mouse anti-PD-L1 monoclonal antibody (1:40 dilution; clone number 22C3; DAKO, Glostrup, Denmark), a mouse anti-FOXP3 monoclonal antibody (1:400 dilution; clone number AB20034; Abcam, MA, USA) and a mouse anti-CD163 monoclonal antibody (1:100 dilution; clone number 10D6; Zhongshan, Beijing, China) at 4°C overnight, using DAKO EnVision detection system (K5007) for immunoassay. Slides were counterstained with Mayer hematoxylin, dehydrated with gradient alcohol, and fixed with neutral resin. Negative control staining was performed with PBS instead of primary antibody.

### Manual Quantification of IHC

Quantification of the IHC stain was analyzed by two pathologists blinded to the clinical characteristics. Five tumor fields at 400x magnification were randomly selected, and agreement was achieved between the observer assessments for each specimen. We evaluated the expression of PD-L1 based on the previously described proportion score (9, 10), using 50% as the cut-off value, and divided TET patients into PD-L1 high expression and low expression group. According to the positive cell rate, the percentage of B7-H4 positive expression was divided into four levels, which are 0 (0%); 1 (1–33%); 2 (34–66%); and 3 (67–100%), the percentage of FOXP3/CD163 positive cell divided into five levels, which were 0 (< 5%); 1 (6–25%); 2 (26–50%); 3 (51–75%) and 4 (> 75%). According to the staining intensity, the positive expression of B7-H4/FOXP3/CD163 was divided into four grades, which are 0 (no staining); 1 (weak staining, light yellow); 2 (mild staining, yellow–brown); and 3 (strong staining, dark brown). The two indicators (staining intensity and percentage of positive cells) were combined to provide a semi-quantitative score (26, 27), and the sum of these two indicators was used to provide the final IHC score, from 0 to 7. According to the IHC score, the tissue staining pattern was defined as low expression (IHC score = 0–2) or high expression (IHC score = 3–7). The IHC analysis has been performed only on the population of this study.

### Evaluating PD-L1 and B7-H4 Co-Expression Patterns

The expression of PD-L1 and B7-H4 was divided into four subgroups: high PD-L1/low B7-H4 expression; high B7-H4/low PD-L1 expression; high B7-H4/high PD-L1 expression (double-high expression); and low B7-H4/low PD-L1 expression (double-low expression). The double-high expression group was defined as “PD-L1/B7-H4 co-expression”, and the other three groups were defined as “No PD-L1/B7-H4 co-expression”. Explore the co-expression pattern of PD-L1 and B7-H4 in different WHO pathological classification.

### Statistical Analysis

This study used Statistical Product and Service Solutions (SPSS) 22.0 statistical software for statistical analyses. The statistical comparison between clinicopatho -logical characteristics and PD-L1/B7-H4 expression was evaluated by the chi-square test, the Fisher’s exact test, and the likelihood-ratio chi-square test (used when necessary). GraphPad Prism 9.0 software was used for recurrence and survival analyses. The starting point was defined as the date of surgical resection. The end of disease-free survival (DFS) was defined as the day of recurrence, and the end of overall survival (OS) was defined as the day of survival or death. The Kaplan–Meier (K-M) method was used to estimate DFS and OS, and the log-rank test was used to compare the curves. P < 0.05 was considered as statistically significant.

## Results

### Differential Expression of PD-L1/B7-H4 in Normal and Tumor Tissues From GTEx and TCGA Database

**Figure 1:**
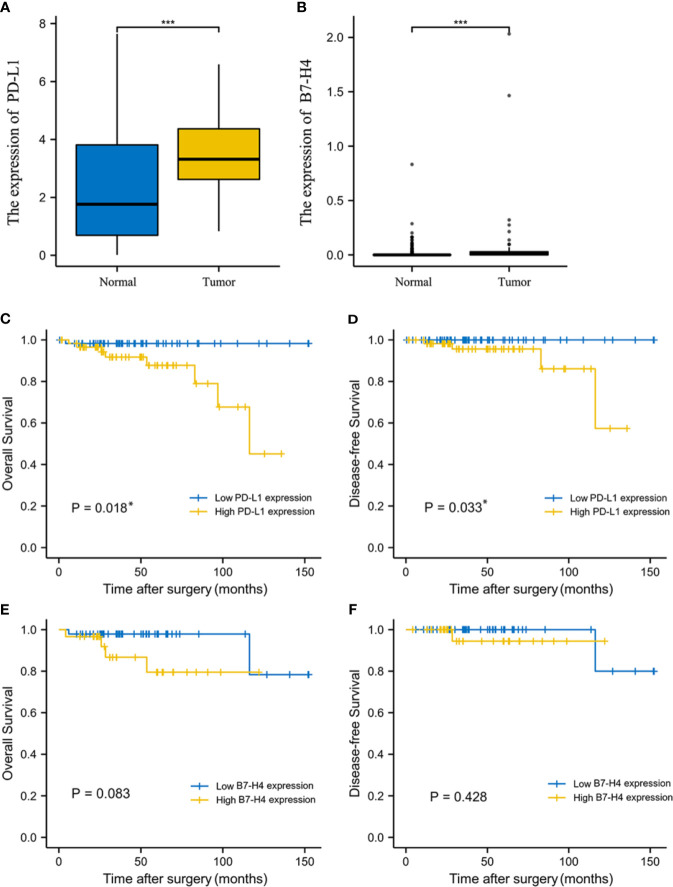
The expression level and prognosis value of PD-L1 and B7-H4 in TCGA database. The expression level of PD-L1 **(A)** and B7-H4 **(B)** between normal tissue and tumor tissue samples from GTEx and TCGA database was significantly difference. Survival analysis of TET patients with high (yellow line) or low (blue line) PD-L1/B7-H4 expression. The high PD-L1 group correlated with poor overall survival (OS, **C**) and disease-free survival (DFS, **D**) in TET. No survival differences, according to B7-H4 expression, were observed in TET **(E, F)**. *P < 0.05, ***P < 0.001.

The comparison of the expression levels of PD-L1 and B7-H4 in normal tissues and TET samples from GTEx and TCGA database was shown in **Figures 1A, B**. It can be found that PD-L1 was expressed in normal tissues, but the expression level in tumor tissues was significantly higher (P<0.001). The expression of B7-H4 in normal tissues and tumor tissues was generally low, but the expression in tumor tissues was significantly higher than that in normal tissues (P<0.001).

### Correlation Between PD-L1/B7-H4 mRNA Expression and Clinicopathological Characteristics of TET Patients From TCGA Database

The cut-off values of PD-L1 and B7-H4 expression were determined by survival receiver operating characteristic (ROC) package. Consequently, the topest 50% high value of PD-L1 was identified as the high PD-L1 expression and the topest 20% high value of B7-H4 was identified as the high B7-H4 expression.

Patients with high PD-L1 expression showed higher frequency of myasthenia gravis symptoms (P = 0.015, **Table 1**). PD-L1 expression had no significant correlation with other clinical characteristics (P > 0.05, **Table 1**).

**Table 1:** Correlation between PD-L1 and B7-H4 mRNA expression with clinicopathological characteristics of TET patients from TCGA database.

Patients clinical characteristic	PD-L1 expression		*P*	B7-H4 expression		*P*
	Low (n = 60)	High (n = 61)		Low (n = 103)	High (n = 18)	
**Age**(year)						
≤60	30	29	0.465	49	10	0.200
>60	30	32		54	6	
**Sex**						
Female	27	31	0.323	50	8	0.385
Male	33	30		53	10	
**Masaoka stage**						
I/II	51	46	0.128	82	15	0.564
III/IV	8	14		19	3	
**WHO pathologic classification**						
A/B	54	55	0.752	97	13	0.003^*^
C	6	5		6	5	
**Myasthenia gravis**						
Present	11	23	0.015^*^	6	8	<0.001^**^
Absent	49	38		97	12	
**Recurrence**						
Yes	10	10	0.581	18	2	0.392
No	50	51		85	16	
**Prognosis**						
Dead	2	7	0.136	6	3	0.252
Alive	58	53		96	15	
Lost to follow-up	0	1		1	0	

*P < 0.05; ^**^P < 0.01.

Patients with high B7-H4 expression had a had a more advanced level of TET (according to the WHO pathological classification) compared to tumors with low B7-H4 expression. The expression of B7-H4 in patients with type C (WHO pathological classification was divide into A/AB/B1/B2/B3/C) was higher than that of patients with type A/B (P = 0.045, **Table 1**). Similarly, patients with high B7-H4 expression showed higher frequency of myasthenia gravis symptoms (P < 0.001, **Table 1**). The expression of B7-H4 was not significantly correlated with other clinical features (P > 0.05, **Table 1**).

### Survival Analysis in TET Patients From TCGA Database

K-M survival analysis showed that PD-L1 mRNA expression levels were related to the prognosis of TET patients. High PD-L1 expression was associated with significantly shorter OS and DFS compared to patients with low PD-L1 expression (P = 0.018, 0.033; **Figures 1C, D**), suggesting that high PD-L1 expression is an indicator of poor prognosis. Patients with high B7-H4 expression had relatively short OS and DFS, but we did not observe a significant difference between B7-H4 high and low expression groups (P > 0.05; **Figures 1E, F**).

### Relationship Between PD-L1/B7-H4 mRNA Expression and Tumor-Infiltrating Immune Cells

**Figure 2:**
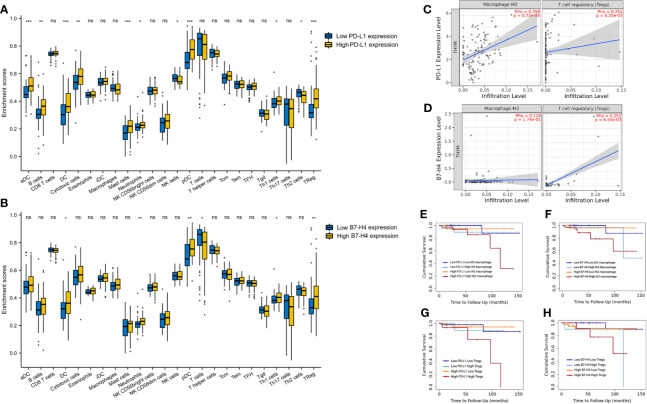
The correlation between PD-L1/B7-H4 and the tumor-infiltration immune cells. The distribution of tumor-infiltrating immune cells in the high and low expression groups of PD-L1 **(A)** and B7-H4 **(B)** was significantly different in TET. A positive correlation existed between the PD-L1 expression level and infiltrating levels of M2 macrophages and Tregs, the B7-H4 expression level and infiltrating levels of Tregs in TET **(C, D)**. Double high groups (red line, high PD-L1 high M2 macrophages; high PD-L1 high Tregs; high B7-H4 high M2 macrophages; high B7-H4 high Tregs) had the worst prognosis compared to the low expression groups **(E–H)**. ns, no significance; *P < 0.05, **P < 0.01, ***P < 0.001.

**Figure 2A** showed that the distribution of tumor-infiltrating immune cells in the high and low expression groups of PD-L1 was significantly different in TET. Particularly, patients with high PD-L1 expression had higher aDC, B cells, cytotoxic cells, DC, mast cells, neutrophils, pDC, Tgd and Tregs (P < 0.05). In contrast, patients with low PD-L1 expression had higher NK cells and Th2 cells (P < 0.05).

A similar situation was observed between the high and low B7-H4 expression groups (**Figure 2B**). Patients with high B7-H4 expression had higher DC, neutrophils, pDC, Tgd, and Tregs compared with low expression (P < 0.05). In contrast, patients with low B7-H4 expression had higher T cells (P < 0.05).

### The Correlation Between the Expression of PD-L1/B7-H4 and the Infiltration of M2 Macrophages/Tregs and Their Combined Prognostic Value

We conducted subsequent analysis on suppressive immune infiltrating cells including M2 macrophages and Tregs. A positive correlation existed between the expression level of PD-L1 and infiltrating levels of M2 macrophages (r = 0.358, P = 8.72e-05) and Tregs (r = 0.254, P =6.25e-03) in TET (**Figure 2C**). Besides, a positive correlation between the expression level of B7-H4 and infiltrating levels of Tregs (r = 0.254, P =6.25e-03) was also explored in TET (**Figure 2D**).

Due to the limitations of a single marker for prognosis, we combined immunosuppressive molecules with tumor immune-infiltrating cells for the prognosis of TET, *via* TIMER 2.0 online tool. According to the expression level of PD-L1/B7-H4 and M2 macrophages/Tregs, it can be divided into four groups. It was obvious that there were significant differences in prognosis among the four groups (**Figures 2E–H**). Among them, we can see that patients with high PD-L1 expression and high M2 macrophages infiltration have the worst prognosis, compared to patients with low PD-L1 expression and low M2 macrophages infiltration (P<0.05). Patients with high PD-L1 expression and high Tregs infiltration also showed a worst prognosis (P<0.05). Similar results were found in B7-H4. Patients with high B7-H4 expression and high M2 macrophages or Tregs infiltration had the worst prognosis compared to the patients with low B7-H4 expression and low M2 macrophages or Tregs (P<0.05).

### Clinicopathologic Characteristics of TET Patients in Clinical Cases

All patient characteristics were presented in **Table 2**. The retrospective analysis was followed up to December 1, 2020, and the median follow-up time was 79 months (range = 12–156 months). A total of 37 men (46.25%) and 43 women (53.75%) were included, with a median age of 60 years (range = 30–79 years). Seventy-seven patients (96.25%) completed surgical resection. Three of the patients could not be resected due to local progression or tumor metastasis and consequently received chemotherapy. In all cases of complete resection, surgical margins were negative under the microscope. Among the enrolled 80 TET patients, 10 (12.5%) patients relapsed during the follow-up period. With respect to prognosis, 14 (17.5%) patients died of TET and 7 (8.75%) patients were lost during follow up.

**Table 2:** Thymic epithelial tumor patient characteristics.

Characteristic	Number of patients (n = 80)	Proportion of patients/%
**Age (year**)		
≤60	37	46.25
>60	43	53.75
**Sex**		
Female	40	50.00
Male	40	50.00
**Masaoka stage**		
I	40	50.00
II	10	12.50
IIIa	23	28.75
IIIb	3	3.75
IV	1	1.25
**WHO pathologic classification**		
A	13	16.25
AB	11	13.75
B1	20	25.00
B2	15	18.75
B3	12	15.00
C	9	11.25
**Myasthenia gravis**		
Present	23	28.75
Absent	57	71.25
**Recurrence**		
Yes	10	12.50
No	70	87.50
**Prognosis**		
Dead	14	17.50
Alive	59	73.75
Lost to follow-up	7	8.75

### The Expression Pattern of PD-L1, B7-H4, CD163, and FOXP3 in TET Tissues

**Figure 3:**
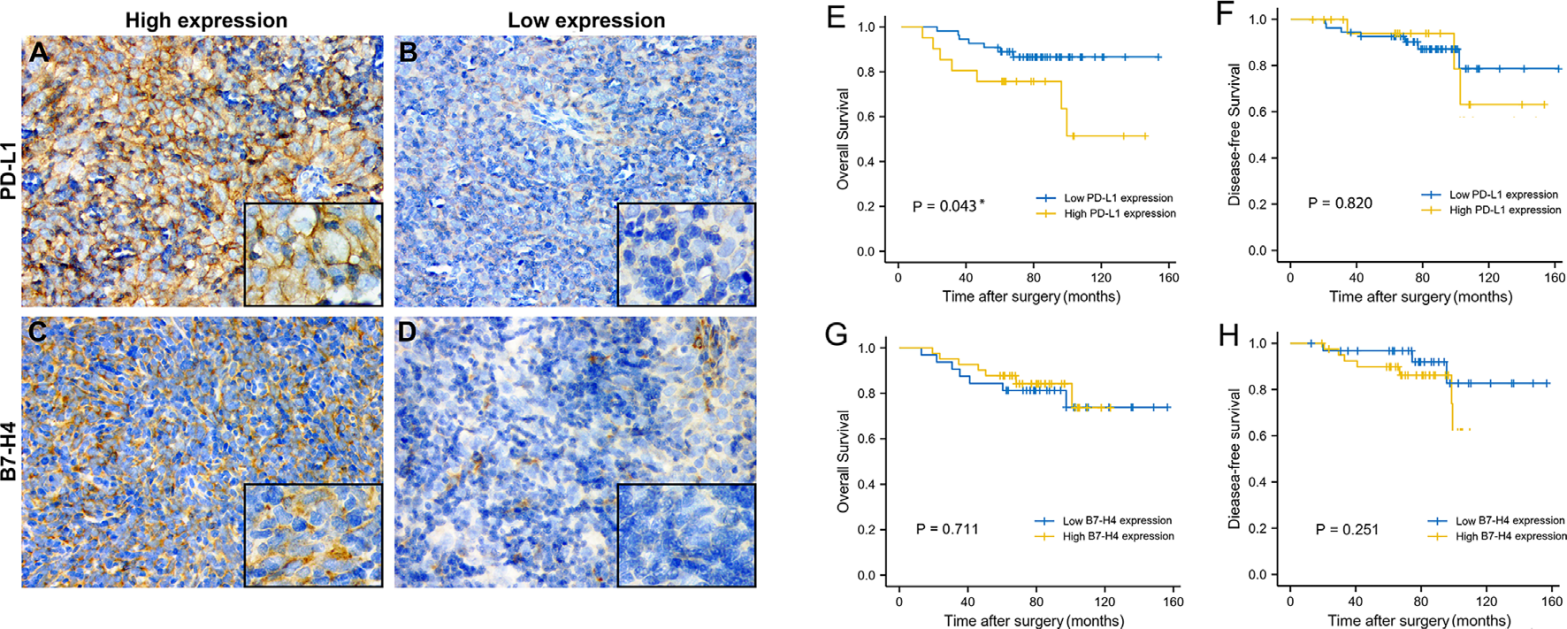
The expression level and prognosis value of PD-L1 and B7-H4 in follow-up TET patients. Representative images of high PD-L1 expression staining (staining score = 3–6) in thymic tumor cells. **(A)**; low PD-L1 expression staining (staining score = 0–2) **(B)**; high B7-H4 expression staining (staining score = 3–6) **(C)**; low B7-H4 expression staining (staining score = 0–2) **(D)**. Original magnification x400 and x1000. Survival analysis of TET patients with high (yellow line) or low (blue line) PD-L1/B7-H4 expression. High PD-L1 expression related to a worse OS **(E)**, but not related to DFS **(F)**. Regardless of OS or DFS, no differences were observed in B7-H4 **(G, H)** expression subgroups. *P < 0.05.

Using immunohistochemical techniques to stain tumor tissues, we found that most patients (78.75%) expressed PD-L1. Among the samples, 60 patients were categorized as having high PD-L1 expression and 20 patients were categorized as having low PD-L1 expression. B7-H4 was expressed in 78.75% of all patients. Among these, 45 patients were categorized as having high B7-H4 expression and 35 patients were categorized as having low B7-H4 expression. PD-L1 and B7-H4 protein expression patterns on TET tumor cells appeared to be diffuse in most cases. Under the microscope, PD-L1 and B7-H4 can be observed in the cell membrane, cytoplasm, or both. Representative images were showed in **Figures 3A–D**.

CD163 showed scattered and strongly positive infiltrating distribution on the cell membrane of M2 macrophages in the stroma of TET. Almost all TET stroma had the infiltration of M2 macrophages. Among them, 43.28% showed high expression and 56.72% low expression. FOXP3 showed punctate and moderately positive infiltrating distribution on the nucleus of Tregs in the tumor stroma. 72.06% TET samples expressed FOXP3, of which 41.18% showed high expression and 58.82% low expression. Representative images were showed in **Figures 5A–D**.

### Statistical Association Between PD-L1/B7-H4 and Clinicopathologic Features of TET Patients in Clinical Cases

Patients with high PD-L1 expression had a higher level of WHO pathology compared to patients with low PD-L1 expression. The expression of PD-L1 in patients with type C TET was higher than those who had type A/B TET (P = 0.025, **Table 3**). Patients with high PD-L1 expression had higher Masaoka clinical staging, and PD-L1 expression in patients with stage III/IV TET was higher than that of patients with stage I/II TET (P = 0.003, **Table 3**). PD-L1 expression had no correlation with age, gender, myasthenia gravis and recurrence rate.

**Table 3:** Correlation between PD-L1 and B7-H4 protein expression with clinicopathological characteristics of TET patients in clinical cases.

Characteristic	PD-L1 expression		*P*	B7-H4 expression		*P*
	Low (n = 60)	High (n = 20)		Low (n = 35)	High (n = 45)	
**Age**						
≤60	28	9	0.190	15	22	0.378
>60	32	11		20	23	
**Sex**						
Female	30	10	1.000	21	19	0.088
Male	30	10		14	26	
**Masaoka stage**						
I/II	43	7	0.003^*^	26	24	0.049^*^
III/IV	15	12		8	19	
**WHO pathologic classification**						
A/B	56	15	0.025^*^	34	37	0.036^*^
C	4	5		1	8	
**Myasthenia gravis**						
Present	14	9	0.064	12	11	0.500
Absent	46	11		23	24	
**Recurrence**						
Yes	7	3	0.696	3	7	0.235
No	53	17		35	38	
**Prognosis**						
Dead	9	5	0.513	7	7	0.874
Alive	45	14		25	34	
Lost to follow-up	6	1		3	4	

*P < 0.05.

Patients with high B7-H4 expression had a higher Masaoka clinical staging compared to patients with low B7-H4 expression. The expression of B7-H4 in stage III/IV patients was higher than in stage I/II patients (P = 0.049, **Table 3**). The expression of B7-H4 in patients with type C TET was higher than those who had type A/B TET (P = 0.036, **Table 3**). The expression of B7-H4 had no correlation with age, gender, myasthenia gravis symptoms, recurrence rate and survival rate.

### Co-Expression of PD-L1 and B7-H4 Across TET Pathological Subtypes

**Figure 4:**
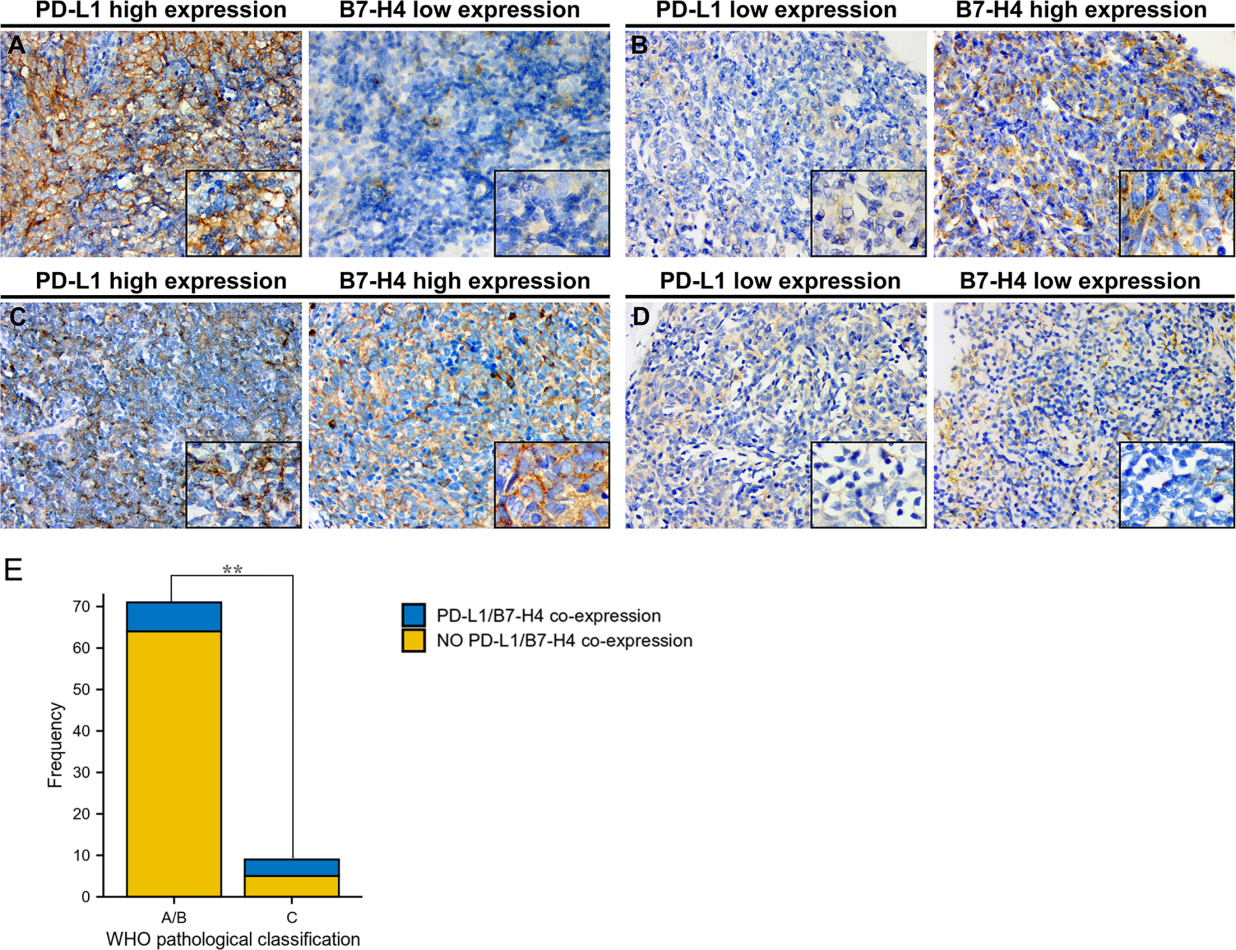
Co-expression of PD-L1 and B7-H4 and distribution of co-expression subgroups in WHO pathological classification of TET. Representative staining images of PD-L1/B7-H4 subgroups were shown with the same area for every two matched samples. High PD-L1/low B7-H4 expression in thymic tumor cells **(A)**; low PD-L1/high B7-H4 expression **(B)**; double-high expression **(C)**; and double-low expression **(D)**. Original magnification x400 and x1000. Both in type A/B and type C of pathological classification, co-expression of PD-L1 and B7-H4 expression was limited. However, compared to patients with low-grade tumor (type A/B), patients with high-grade tumor (type C) harbored higher co-expression of PD-L1 and B7-H4 (P = 0.005, **E**). **P < 0.01.

We observed that high PD-L1/low B7-H4 expression and high B7-H4/low PD-L1 expression are the main expression patterns in TET (**Table 4**). It can be seen that PD-L1 is negatively correlated with B7-H4 in most cases, including patients having low-grade (90.1%) or high-grade (55.6%) TET (determined by the WHO pathological classification). Representative images were showed in **Figures 4A–D**.

**Table 4:** Distribution of PD-L1/B7-H4 expression subgroups in the different WHO pathological classification groups.

PD-L1/B7-H4 subgroup	Number of patients with A/B classification (proportion of total number of patients/%)	Number of patients with C classification (proportion of total number of patients/%)
Double-high expression	7 (9.9%)	4 (44.4%)
Double-low expression	26 (36.6%)	0 (0.00%)
High PD-L1/Low B7-H4 expression	8 (11.3%)	1 (11.1%)
High B7-H4/Low PD-L1 expression	30 (42.3%)	4 (44.4%)

However, as TET progressed, the percentage of patients co-expressing PD-L1 and B7-H4 raised from 9.9% to 44.4% (**Table 5**), which showed a significant difference between low-grade and high-grade tumors (determined by the WHO pathological classification; P = 0.005; **Figure 4E**).

**Table 5:** Co-expression of PD-L1/B7-H4 subgroups in WHO pathological classification of TET.

PD-L1/B7-H4 subgroup	A/B	C
PD-L1/B7-H4 co-expression	7 (9.9%)	4 (44.4%)
No PD-L1/B7-H4 co-expression	64 (90.1%)	5 (55.6%)

### Survival Analysis of TET Patients in Clinical Cases

The OS and DFS of PD-L1 and B7-H4 expression in TET were shown in **Figure 3**. With the day of resection as the starting point, the end of DFS was defined as the day of relapse and the end of OS was defined as the day when survival or death was confirmed. K-M survival analysis showed that high PD-L1 expression was positively related to the shorter OS of patients (P = 0.043, **Figure 3E**). B7-H4 expression levels had no significant correlation with the OS of patient but as can be seen from **Figure 3G**, patients with low B7-H4 expression have shorter OS and may have a poorer prognosis than high B7-H4 expression. PD-L1 and B7-H4 have no correlation with a patient’s DFS (P > 0.05, **Figures 3F, H**)

### Correlation Between PD-L1/B7-H4 Expression and FOXP3/CD163 Infiltration and Combined Prognostic Value

The expression of PD-L1 was consistent with CD163 and FOXP3, which meant that when PD-L1 is highly expressed, CD163 and FOXP3 are also highly expressed, and the difference is statistically significant (P=0.029, P=0.004). As for the expression of B7-H4 and CD163, 47.76% showed consistency, but there was no significant difference (P = 0.483). The expression of B7-H4 was consistent with FOXP3. When B7-H4 was highly expressed, FOXP3 was also highly expressed, and the difference was statistically significant (P = 0.037).

**Figure 5:**
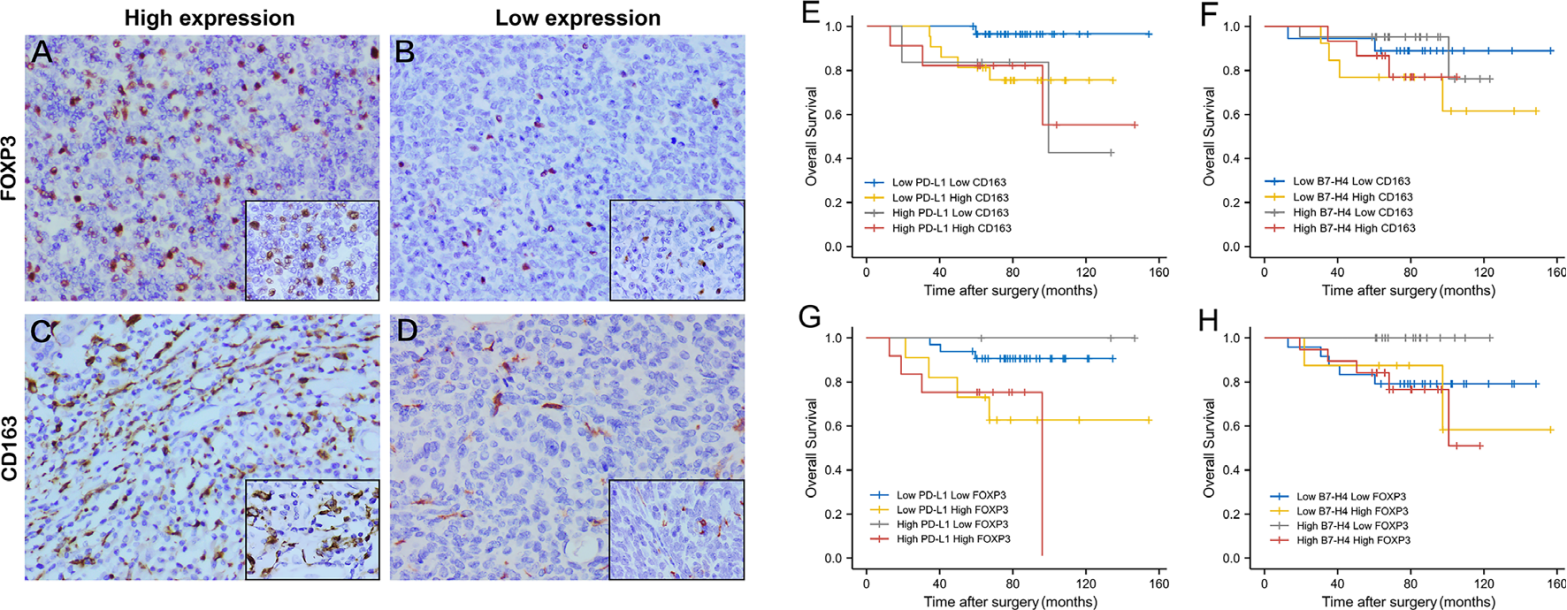
The expression level and combined prognosis value of FOXP3 and CD163 in follow-up TET patients. Representative images of high FOXP3 expression staining (staining score = 3–7) in thymic tumor cells. **(A)**; low FOXP3 expression staining (staining score = 0–2) **(B)**; high CD163 expression staining (staining score = 3–7) **(C)**; low CD163 expression staining (staining score = 0–2) **(D)**. Original magnification x400 and x1000. Survival analysis of TET patients combined PD-L1/B7-H4 and FOXP3/CD163 expression. High PD-L1 High CD163 expression had a worst prognosis compared to patients with low expression. **(E)** But the combination of B7-H4 and CD163 had a poor prognostic effect. **(F)** High PD-L1 High FOXP3 expression had the worst prognosis compared to patients with low expression. **(G)** Patients with high expression of B7-H4 and high expression of FOXP3 also had the worst prognosis **(H)**.

It can be seen from the K-M survival curve that patients with high PD-L1 and high CD163 expression have a significant difference in prognosis compared with patients with low PD-L1 and low CD163 expression (P = 0.031, **Figure 5E**). The combination of B7-H4 and CD163 had a poor prognostic effect, but it can be found that the two groups of patients with high expression of FOXP3 (Low B7-H4 High FOXP3/High B7-H4 High FOXP3) have a worse relative prognosis (**Figure 5F**).

Similar to the combination of PD-L1 and CD163, patients with high PD-L1 expression and high FOXP3 expression had the worst prognosis compared with low PD-L1 expression and low FOXP3 expression (P = 0.026, **Figure 5G**). Patients with high expression of B7-H4 and high expression of FOXP3 also had the worst prognosis (P = 0.028, **Figure 5H**).

## Discussion

In this study, we found that B7-H4 mRNA expression was positively related to the WHO pathological classification from TCGA database, suggesting that B7-H4 expression can indicate the aggressiveness of TET. In addition, we found that PD-L1 mRNA levels were positively related to the clinical outcome (death or recurrence) of patients.

Since the TCGA database focused on mRNA expression levels, we also assessed the protein expression levels of PD-L1 and B7-H4 in 80 TET patients using immunohistochemistry, confirming the expression and localization of PD-L1 and B7-H4 in tumor tissues. The results showed that most patients with TET express PD-L1 and B7-H4, and high PD-L1 and B7-H4 expression indicated high-grade TET progression. Survival analysis showed that high PD-L1 expression can predict poor OS but B7-H4 were not effective in predicting the survival and recurrence of patients with TET. From the TCGA database, PD-L1 mRNA expression was detected in almost all patients with TET, whereas B7-H4 mRNA expression was only detected in 58.1% of patients. But PD-L1 and B7-H4 protein expression levels were similar. This may be due to differences caused by the translation process of mRNA to protein or deviation caused by the immunohistochemical detection of protein.

In recent years, the rapid development of immunotherapy has confirmed that antibody therapy targets PD-1/PD-L1 or disrupts the interaction of PD-1/PD-L1 has shown good therapeutic effects in many solid tumors (28). Studies have shown that TET is one of the rarest tumor types with the highest positive rate of PD-L1 (29). Although PD-L1 is also expressed in normal thymic cortical cells, it is highly expressed in TETs (30), which can provide a theoretical basis for the implementation of immunotherapy to treat TET. According to previous studies, PD-L1 expression is related to high-grade Masaoka staging, WHO pathological classification, or both (11–13, 31, 32). The results of this study showed that patients with high-grade TET (type C) have higher PD-L1 expression than patients with type A/B TET, which was consist with the results of several studies.

We reviewed and summarized 17 published research results on PD-L1 expression in TET, which were listed in **Table 6** (9–14, 31–41). From these studies, six studies showed that PD-L1 expression is related to Masaoka stage (9, 11–13, 32, 41) and 10 studies showed that PD-L1 expression is related to WHO pathological classification (9–13, 32, 36, 40, 41). However, the data for the prognostic significance of PD-L1 for TET was limited and had conflicting results. Most of these studies did not report the difference in OS and DFS between the high and low PD-L1 expression groups. There are three studies (9, 12, 13) that reported thymoma with high PD-L1 expression had worse OS, and one study (14) that showed that the high PD-L1 expression group had better OS. The results of this study were consistent with the study of Hakiri S and Padda SK, indicating that patients with high PD-L1 expression had worse OS. But the prognostic significance of PD-L1 is still controversial and needs further investigation.

**Table 6:** Summary of published studies investigating PD-L1 expression in TET.

Reference	Number of TET patients	PD-L1 cut-off	PD-L1 positivity in TET	Significant characteristic in TET(For PD-L1 high expression group)	Prognostic value(For PD-L1 high expression group)
9	308	≥50%, proportion	279 (90.6%)	High Masaoka stage, high WHO classification, and myasthenia gravis	Poor OS and DFS
10	100	Strong intensity or ≥50% in moderate intensity	36 (36%)	High masaoka stage and type B histology	No significance in OS and DFS
11	32	≥3, Semiquantified(0–5)	26 (81%)	None	No significance in OS and DFS
12	82	≥38%, proportion	44 (53.7%)	Type B2 and B3 histology and high Masaoka stage (III and IV)	No significance in OS and DFS
13	50	≥3 (1%), H-score	24 (48.0%)	High WHO classification	Not evaluated
14	12	≥25%, TPS	11 (91.7%)	None	Good OS
31	81	≥1%, TPS	22 (27.0%)	Type B2 and B3 histology, high stage (III and IV)	Poor OS
32	74	≥5%, TPS	47 (64%)	Neoadjuvant chemotherapy	No significance in OS and DFS
33	38	≥1, Semiquantified(0–3)	35 (92%)	None	Not evaluated
34	84	≥25%, TPS	69 (82.1%)	Type B histology	No significance in OS and DFS
35	31	≥1%, TPS	16 (51.6%)	WHO pathological classification	Not evaluated
36	13	≥6, Semiquantified(0–12)	6 (46.2%)	None	No significance in OS
37	12	≥1, H-score	6 (67%)	None	No significance in OS
38	29	≥50%, TPS	9 (31.0%)	Low Masaoka stage	No significance in OS and DFS
39	63	≥1%, TPS	45 (74%)	PD-L1 mRNA over-expression, tumor diameter, locally advanced TETs	Not evaluated
40	38	≥5%, TPS	31 (81.5%)	Type B3 histology	No significance in OS and DFS
41	65	Score 3, intensity(0–3)	44 (68%)	Younger age, high Masaoka stage, incomplete resection, and aggressive histology	Poor OS

H-score, histochemistry score; TPS, tumor proportion score; OS, overall survival; DFS, disease-free survival; TETs, thymic epithelial tumors.

As an important co-stimulatory molecule of the B7 family, B7-H4 (VTCN1) inhibits the response of T cells by interacting with unknown receptors on the surface of T cells, thereby mediating tumor immune escape (42). Immunotherapy targeting B7-H4 has gradually become an important adjuvant therapy to traditional chemotherapy in several solid tumors. Studies have shown that immunotherapy helps tumor regression and the reduction of recurring lesions (10). Whether B7-H4 as an immune checkpoint can help promote the application of immunotherapy in TET is what we want to explore.

Lingwei Shen et al. (15) showed that B7-H4 is highly expressed in pancreatic cancer and is related to tumor Tumor Node Metastasis (TNM) staging and distant metastasis and demonstrated high B7-H4 expression is relevant to the poor prognosis of pancreatic cancer; Nah Ihm Kim et al. (17) explored the correlation between the expression of B7-H3 and B7-H4 and the clinicopathological characteristics of breast cancer. Although the results showed that there was no association between expression levels and survival, the expression of B7-H4 is inversely related to the density of stromal Tumor Infiltrating Lymphocytes (TILs) and cluster of differentiation (CD) 8 T lymphocytes. This inverse relationship may suggest that B7-H4 can serve as a promising target in the field of breast cancer immunotherapy. In addition, studies have shown that B7-H4 is associated with pathological classification and clinical features of ovarian cancer (16), endometrial cancer (43), and hepatocellular carcinoma (44), such as tumor size, tumor stage, progression, and TIL infiltration. To the best of our knowledge, we are the first group to report B7-H4 expression patterns in TET. Perhaps the effect of B7-H4 expression in promoting TET to evade immune surveillance and causing adverse immune outcomes is not decisive, but it may have significance in adjuvant treatment and prognosis as an immune checkpoint. Our study showed that B7-H4 is expressed in 78.75% of TET patients; B7-H4 expression levels were found to be associated with patient clinicopathological characteristics and could identify type C patients. We look forward to observing more research on B7-H4 expression in TET and the clinical application of B7-H4 as a target in immunotherapy.

We also specifically studied the potential association between PD-L1 and B7-H4 co-expression level with the specific WHO pathological classification of TET. Previous studies (45) showed that PD-L1 and B7-H4 expression in gliomas exhibit a mutually exclusive pattern, lacking a double-high expression subgroup. Our results confirmed that the co-expression pattern of PD-L1 and B7-H4 may indicate different stages of clinical progression of TET. Overall, most tumors exhibited a negative correlation between their PD-L1 and B7-H4 expression, but the co-expression of PD-L1 and B7-H4 increased in high-grade TET. One possibility is that either PD-L1 or B7-H4 mediates immunosuppression in early stages of TET, but both contribute to immune evasion as the disease progresses. Our findings can be used as a potential anti-TET therapeutic strategy, suggesting that the combined targeting of PD-L1 and B7-H4 may be able to overcome the current limitations of single immune checkpoint therapy.

Our study first explored the expression level and clinical significance of immunosuppressive molecules including PD-L1 and B7-H4. In the following research, we focused on the relationship between immunosuppressive molecules and tumor immune-infiltrating cells in TME of TET. TME is a complex network formed by the interaction between the immune system and tumor cells. A more in-depth analysis of TME may reveal advanced biomarkers (46, 47). In particular, tumor-specific immune infiltrating cells may be essential for tumor signaling and predicting the prognosis of patients. So we analyzed the infiltration of immune cells in TME. With the continuous advancement of biological information technology, deconvolution methods using RNA-seq data (such as quanTIseq, xCell, CIBERSORT, ssGSEA, etc.) can be used to locate TME and evaluate immune infiltration (23, 48). We first used the ssGSEA algorithm to analyze 24 kinds of immune infiltrating cells and found that there were indeed differences in the TME between high and low PD-L1/B7-H4 groups.

Owing to technical limitations, we only classified 24 types of immune cells, including the macrophages level, but did not further classify and evaluate the infiltration level of M0/M1/M2 macrophages. As previous studies have shown, M2 macrophages were considered essential immune cells and play a key role in tumor growth, angiogenesis and metastasis (49, 50). Therefore, we targeted M2 macrophages for subsequent analysis. In addition, Tregs play a significant role in maintaining the stability of the immune system, and tumor immune tolerance and escape due to an immunosuppressive ability (51). A large number of studies have shown that Tregs is associated with poor prognosis (52, 53). Since the immune infiltration analysis of TET showed that Tregs were significantly different in the high and low PD-L1 and B7-H4 groups, we conducted a follow-up analysis of Tregs.

With the help of the online tool TIMER2.0, we found that PD-L1 is positively correlated with M2 macrophages and Tregs, and B7-H4 is positively correlated with Tregs. Since our preliminary study showed that the prognostic effect of PD-L1 and B7-H4 alone is not ideal, we tried to combine M2 macrophages and Tregs for prognostic analysis. We found that high expression of PD-L1 and M2 macrophages or Tregs, high expression of B7-H4 and Tregs is associated with poor prognosis in TET patients. It can be seen that the combination of immunosuppressive molecules and immune infiltrating cells can better guide the prognosis of patients to a certain extent.

We used FOXP3 and CD163 as protein markers to specifically identify Tregs and M2 macrophages, respectively, through immunohistochemistry. We observed that the expression of FOXP3 and CD163 in the high PD-L1 expression group was higher than that in the low PD-L1 expression group. The analogous phenomenon was also found in high B7-H4 expression group, indicating the importance of PD-L1 and B7-H4 in the suppressive tumor immune microenvironment. After prognostic analysis with PD-L1/B7-H4 combined with FOXP3/CD163, it was found that the double high group (high PD-L1 high CD163, high PD-L1 high FOXP3 and high B7-H4 high FOXP3) shortened overall survival time, confirming that the inhibitory tumor microenvironment led to poor prognosis.

Overall, comprehensive evaluation of the expression level of immune examination molecules in TET patients and the distribution of immune infiltrating cells in the tumor microenvironment can provide a reference for the formulation of personalized immunotherapy programs. Targeting immunosuppressive molecules while regulating the tumor microenvironment may provide new ideas for the treatment of tumor patients and have an important impact on clinical management.

This study faces some limitations. Firstly, standardization of the staining intensity scores for PD-L1 and B7-H4 in TETs have not yet been developed, potentially leading to differences in scoring standards between studies and results. Secondly, the number of cases included in our single-center retrospective study was relatively small. Due to the few survival events in our study, it is difficult to correct the potential value of PD-L1 and B7-H4 as TET survival markers for meaningful analysis or determine the extent of PD-L1 and B7-H4 as prognostic factors. Therefore, longer follow-up time may be needed to assess the prognostic value of PD-L1 and B7-H4. Thirdly, a variety of deconvolution algorithms have been developed for tumor background prediction, but the results of each method vary a lot, and there is no “gold standard” method for analyzing immune infiltrating cells in TME. This article only uses two methods for evaluation and more studies are needed in the future to evaluate the relationship between PD-L1/B7-H4 and immune infiltration of TET.

## Conclusion

In summary, we conducted a comprehensive bioinformatics analysis and IHC assessment on TET patients. We confirmed PD-L1 and B7-H4 can serve as potential markers of TET aggressiveness and their obvious correlation with the infiltration of TIICs. We demonstrated the significance of the expression of B7-H4 and PD-L1 combined with the infiltration of M2 macrophages and Tregs on the TET clinical outcome. Considering the complex network that constitutes the tumor microenvironment, such comprehensive analysis should be performed when formulating immunotherapy methods and evaluating the response of tumor patients.

## Data Availability Statement

The original contributions presented in the study are included in the article/supplementary material. Further inquiries can be directed to the corresponding author.

## Ethics Statement

The studies involving human participants were reviewed and approved by Ethics Committee of the Second Affiliated Hospital of Zhejiang University School of Medicine. The patients/participants provided their written informed consent to participate in this study. Written informed consent was obtained from the individual(s) for the publication of any potentially identifiable images or data included in this article.

## Author Contributions

XY and JF worked on data analysis and interpretation. XY drafted the manuscript. BH collected specimens of thymic epithelial tumor tissues and carried out the IHC staining experiments. YQ designed the study. All authors contributed to the article and approved the submitted version.

## Funding

This work was supported by the National Natural Science Foundation of China [grant number 81802081]. The funders had no role in study design, data collection and analysis, decision to publish, or preparation of the manuscript.

## Conflict of Interest

The authors declare that the research was conducted in the absence of any commercial or financial relationships that could be construed as a potential conflict of interest.
